# Megacolon due to Chronic Schistosomiasis: A Case Report and Review of Literature

**DOI:** 10.1155/2019/4036823

**Published:** 2019-03-06

**Authors:** Amer R. Alzahrani, Homoud Alawfi, Sara Almeman, Thamer Altayeb, Hasan M. Al-Dorzi

**Affiliations:** ^1^Surgery Department, King Abdulaziz Medical City, King Saud bin Abdulaziz University for Health Sciences and King Abdullah International Medical Research Center, Riyadh, Saudi Arabia; ^2^Anatomical Pathology Department, King Abdulaziz Medical City, King Saud bin Abdulaziz University for Health Sciences and King Abdullah International Medical Research Center, Riyadh, Saudi Arabia; ^3^Intensive Care Department, King Abdulaziz Medical City, King Saud bin Abdulaziz University for Health Sciences and King Abdullah International Medical Research Center, Riyadh, Saudi Arabia

## Abstract

Although Schistosoma infection in humans commonly involves the intestines, megacolon is a rare finding. We report a 47-year-old patient who was found to have chronic megacolon. After failing conservative management, he underwent extended hemicolectomy with colorectal anastomosis. The colon pathology revealed chronic schistosomiasis and Schistosoma serology was positive.

## 1. Introduction

Megacolon can be defined as the irreversible dilatation of a colonic segment in the absence of obstruction [1]. Although controversial, a cecal diameter ≥ 12 cm is usually used as a cut-off for diagnosis [2]. The acute form can be toxic and is usually associated with severe inflammatory or infectious colonic disease or nontoxic, such as with Ogilvie's syndrome [1]. Chronic megacolon is rare in adults and is commonly idiopathic [1, 3]. However, it may be associated with Chagas' disease and disorders affecting the intestinal smooth muscles or enteric nervous system, which may include spinal cord myelopathy [1, 4]. Even though being very uncommon, Hirschsprung's disease may present in adulthood with chronic megacolon [1].

The intestines are frequently involved during Schistosoma infection especially with Schistosoma mansoni [5, 6]. In this report, we present a case of chronic megacolon associated with colonic schistosomiasis, which has not been reported in literature to our knowledge.

## 2. Case Report

**Figure 1:**
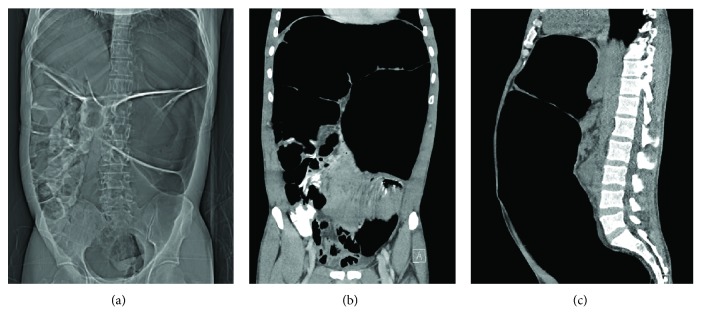
Preoperative computed tomography of the abdomen. (a) The initial scout film shows severely dilated sigmoid colon. (b) Coronal section. (c) Sagittal section.

A 47-year-old man known to have hypothyroidism and hypertension on treatment presented to the gastroenterology clinic complaining of two-year history of abdominal distension that was worse after oral intake especially milk. He had normal bowel motions and denied nausea and vomiting. There was no previous abdominal surgery. On examination, the abdomen was distended with no tenderness. Colonoscopy was done and showed normal rectum, grossly dilated sigmoid with redundant colonic wall, and mild mucosal inflammation. Abdomen computerized tomography showed distended sigmoid colon with collapsed rectum and no obstruction (Figure 1).

**Figure 2:**
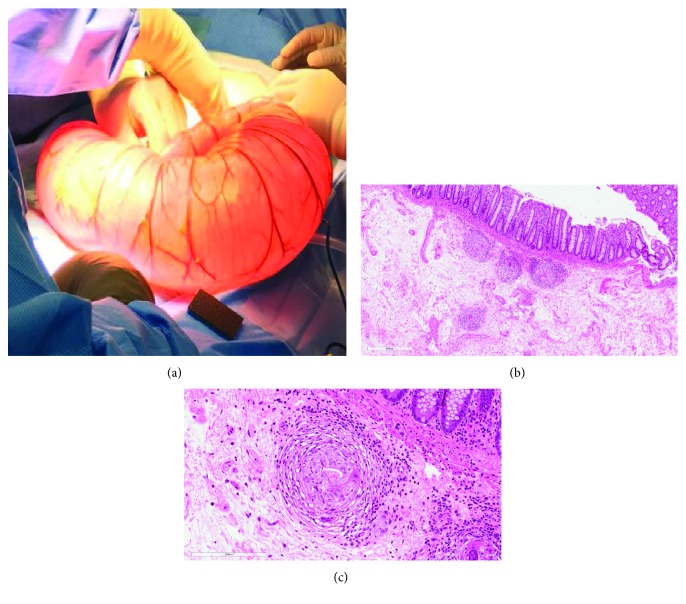
(a) Megacolon as seen at the time of laparotomy. (b) Submucosal granuloma around eggs in the colonic wall (low power). Colonic mucosa shows chronic inactive colitis, mild with mild crypt distortion. There was no dysplasia or malignancy. (c) Submucosal granuloma around eggs in the colonic wall (high power).

As his symptoms were severe and he already failed conservative management, the patient was referred to general surgery. He underwent laparotomy (Figure 2(a)) and extended hemicolectomy of the affected segment, with colorectal anastomosis. He did well intra- and postoperatively. The pathology unexpectedly showed chronic schistosomiasis in the colonic wall (Figures 2(b) and 2(c)). Upon further evaluation, we found that he lived in the north of Saudi Arabia (Hail) but denied exposure to unclean water or recent travel. His Schistosoma serology titer was high (1 : 1024). Other laboratory findings included slightly elevated direct and total bilirubin (12.3 and 41.5 *μ*mol/L, respectively), normal aminotransferases, erythrocyte sedimentation rate = 11 mm/hour, and C-reactive protein < 3.50 mg/L. He was referred to the infectious disease clinic and was treated with praziquantel. At six-month follow-up, the patient was doing well with resolution of his abdominal symptoms.

## 3. Discussion

We reported the case of colonic schistosomiasis associated with chronic megacolon. Review of literature did not yield any similar case.

Chronic megacolon usually manifests as constipation [1]. It is most commonly idiopathic [3]. O'Dwyer et al. reviewed electronic medical records of all patients diagnosed with chronic megacolon from 1999 to 2014 at Mayo Clinic and found that the cause of megacolon was idiopathic in 16 (66.7%) patients [3]. Evaluation of chronic megacolon usually consists of colonoscopy and radiologic studies [1]. If there is travel or living in South America, serologic studies for Chagas' disease are warranted [1]. In a young male with constipation since childhood, Hirschsprung's disease should be considered [1].

The management of megacolon is usually symptomatic and nonsurgical [1]. Addressing the cause, whenever reversible, is crucial. Bowel cleansing with enemas should be done in case of large stool retention [1]. Fiber restriction with small amounts of PEG solutions to decrease stool volume and gas formation is a part of maintenance therapy [1]. If conservative measures fail, surgery may be indicated [1].

Schistosomiasis is a common chronic helminth disease caused by the Schistosoma trematode worms. It starts by Schistosome cercariae penetrating the human skin and becoming schistosomulae; schistosomulae then migrate to the portal vein and mature into adults; adults migrate to the veins draining the intestines, rectum, and bladder [6, 7]. Intestinal schistosomiasis occurs as eggs migrate through the intestinal wall, provoking mucosal granulomatous inflammation [7]. Pseudopolyps may form and superficial bleeding may occur [7]. Most lesions are situated in the large bowel and rectum [7]. With time, the inflammatory response to eggs is attenuated [7]. Intestinal schistosomiasis is most commonly seen with Schistosoma mansoni [5, 6]. However, it may also occur with Schistosoma japonicum, hematobium, and intercalatum [5, 6]. Schistosoma mansoni is endemic in Africa, South America, and the Middle East including certain parts of Saudi Arabia [5, 6]. A study from Saudi Arabia evaluated 216 patients with schistosomal colonic disease and found that eight patients had schistosomal polyps and that the most common histopathological finding in the colonic biopsies was Schistosoma mansoni ova in the colonic mucosa with no or mild inflammation [8].

The most common symptoms and signs of intestinal schistosomiasis are chronic or intermittent abdominal pain, anorexia, and diarrhea, which might be bloody [7]. Moreover, Schistosoma infection has been associated with colon cancer (odds ratio = 3.3; 95% confidence interval = 1.8-6.1) [9]. Chronic megacolon has not been reported. In the study from Saudi Arabia, none of the 216 patients with colonic schistosomiasis was diagnosed with megacolon [8]. The mechanisms by which intestinal schistosomiasis causes chronic megacolon are unclear. It is possible that Schistosoma-induced colonic wall inflammation chronically affects the intestinal smooth muscles and/or enteric nervous system leading to chronic megacolon. In support of this possibility, one study showed that Schistosoma mansoni infection attenuated colitis in rats, but the colitis-induced disturbances in contractility of longitudinal and circular colonic muscle strips persisted for a long period after the inflammatory reaction [10].

Our patient had chronic megacolon with significant symptoms despite medical management. He was treated with hemicolectomy. Intestinal schistosomiasis was found on pathology. He was treated with praziquantel after hemicolectomy, which is the drug of choice for schistosomiasis and is effective against all Schistosoma species [7]. It is unknown whether treatment with praziquantel before surgery would have improved the gastrointestinal symptoms of our patient.

In summary, we report an unusual presentation of a chronic megacolon with pathology showing intestinal schistosomiasis after hemicolectomy. Clinicians and pathologists need to be more aware of this diagnosis.
